# Stage of breast cancer at diagnosis among women with cosmetic breast implants

**DOI:** 10.1038/sj.bjc.6600819

**Published:** 2003-03-18

**Authors:** L R Hölmich, L Mellemkjær, K A Gunnarsdóttir, U B Tange, C Krag, S Møller, J K McLaughlin, J H Olsen

**Affiliations:** 1 Danish Cancer Society, Institute of Cancer Epidemiology, Strandboulevarden 49, 2100 Copenhagen, Denmark; 2 Danish Breast Cancer Cooperative Group: DBCG Secretariat, Rigshospitalet, Blegdamsvej 9, 2100 Copenhagen, Denmark; 3 Department of Oncology, Copenhagen University Hospital, Rigshospitalet, Blegdamsvej 9, 2100 Copenhagen, Denmark; 4 Department of Plastic Surgery, Herlev Hospital, University of Copenhagen, Ndr. Ringvej, 2730 Herlev, Denmark; 5 International Epidemiology Institute, 1455 Research Boulevard, suite 550, Rockville, MD 20850, USA; 6 Department of Medicine, Vanderbilt University Medical Center, Vanderbilt-Ingram Comprehensive Cancer Center, Nashville, TN, USA

**Keywords:** augmentation, breast cancer, breast implants, breast cancer diagnosis, breast neoplasms, silicone

## Abstract

Concern has been raised about the potential delay in breast cancer diagnosis in the augmented breast. We linked a cohort of 2955 women, who received cosmetic breast implants in Denmark during the period 1973–1997 with the Danish Cancer Registry and the Danish Breast Cancer Cooperative Group register. We identified 23 incident cases of invasive breast cancer diagnosed subsequent to breast implantation. We randomly selected 11 controls for each case from the Danish Breast Cancer Cooperative Group's register, and obtained detailed information on all study subjects about surgery, histopathology and stage of breast cancer at diagnosis, intended adjuvant treatment according to trial protocols and overall survival. We found that women with breast implants on average were diagnosed with breast cancer at the same stage as controls. Significantly more women with breast implants had tumour cells in the surgical margins according to the Danish Breast Cancer Cooperative Group's data. There was no significant difference in overall survival between the two groups after an average of 6.4 years of follow-up. Based on this limited number of women with breast cancer subsequent to breast augmentation, breast implants do not appear to delay the diagnosis of breast cancer, and no evidence of impaired survival after breast cancer diagnosis in augmented women was found.

Breast implants are widely used for both reconstructive and cosmetic purposes. There is no evidence that breast implants increase the risk of breast cancer (Brinton *et al*, 2000b; Mellemkjaer *et al*, 2000). On the contrary, several reports document that women with cosmetic breast implants seem to be at a lower risk of developing breast cancer (Berkel *et al*, 1992; Deapen *et al*, 1997; McLaughlin *et al*, 1998; Hoshaw *et al*, 2001). However, the issue of potential delay in breast cancer diagnosis in the augmented breast has been raised, since breast implants are radiopaque and this influences the sensitivity of mammography (Eklund *et al*, 1988; Hayes *et al*, 1988; Silverstein *et al*, 1992). Clinical reports have suggested a possible delay and difficulties in the diagnosis of breast cancer (Leibman and Kruse, 1990; Silverstein *et al*, 1990a; Carlson *et al*, 1993; Fajardo *et al*, 1995), but most epidemiological studies have not supported a delay in diagnosis or impaired survival among women with breast implants (Birdsell *et al*, 1993; Deapen *et al*, 2000).

We identified all subsequent incident cases of invasive breast cancer within a large cohort of women with breast implants. The stage distribution at diagnosis, tumour characteristics and overall survival of cohort women with breast cancer were compared with those of a random sample of women with breast cancer from the general population matched to the cohort on age and year of diagnosis. This study entails more detailed and uniformly collected information about tumour stage and characteristics than previous studies.

## MATERIAL AND METHODS

We have previously identified 2740 women, who underwent cosmetic breast implantation before the age of 55 years at private clinics and public hospitals in Denmark during 1973–1995 (Mellemkjaer *et al*, 2000). The women treated in the private clinics were identified from the clinics' files, while the women treated in public hospitals were identified through the National Register of Patients. For each woman, we obtained the date of breast implantation and the Personal Identification number, a unique 10-digit number, which encodes the date of birth and sex of all individuals in Denmark. An additional 192 women who were treated in private clinics during 1996–1997, and 23 women who were over 55 years of age at implantation, were also included in the present study, resulting in a final cohort of 2955 women with cosmetic breast implants.

The cohort was linked to the Danish Breast Cancer Cooperative Group (DBCG) register using the Personal Identification number to identify women with invasive breast cancer subsequent to breast implantation. The DBCG register is a clinical database, established in 1977 and designed primarily to evaluate the programmes for adjuvant trials among women with primary breast cancer. The register receives detailed information on primary surgical procedure, histopathological examination, adjuvant treatment and clinical follow-up. Based on tumour stage and menopause status, the patients are allocated to groups with high or low risk of recurrence, and are treated in protocols accordingly. Furthermore, the information on patients treated outside protocol programmes is recorded (Andersen and Mouridsen, 1988; The DBCG Secretariat, 1998). A comparative study using data from both the Danish Cancer Registry, which is the nationwide compulsory register for all cancers established in 1943, based on reports from clinicians, pathologists and also from death certificates (Storm *et al*, 1997), and the DBCG showed highly consistent data for younger (98%) and middle-aged (95%) women diagnosed with breast cancer, whereas the DBCG had 21% missing cases of breast cancer for the group over 70 years of age (Rostgaard *et al*, 2000).

Women with breast implants were followed for invasive breast cancer from date of implantation or 1 January 1977, whichever occurred last, until the age of 76 years, date of breast cancer diagnosis, date of death, emigration, or 1 October 2001, whichever occurred first. Computer linkage of the study cohort to the DBCG register identified 21 women with cosmetic breast implants, who subsequently developed breast cancer.

We also linked the implant cohort with the Danish Cancer Registry using data available until the end of 1998. We confirmed the 21 cases found in the DBCG and identified additionally two women with cosmetic breast implants who were registered with subsequent breast cancer, but who had not been reported to the DBCG. Their medical files were obtained, and the relevant information for this study was abstracted. Both cases were subsequently reported to the DBCG register.

For each of the 23 breast cancer cases with cosmetic breast implants (cases), we randomly selected 11 controls with invasive breast cancer from the DBCG register, matched on age and calendar year of breast cancer diagnosis 11 was the minimal number of available individually matched controls, and consequently this number of controls was chosen for each case, resulting in a total of 253 control subjects from the background population (controls).

Information on the following variables was obtained from the DBCG register: date of cancer diagnosis, place of operation, menopausal status, type of surgery and assessment of whether the surgical margins were tumour free (side and deep margins). The histopathological and staging variables were number of lymph nodes removed, number of involved lymph nodes, size of tumour (macroscopic size, evaluated by the pathologist), oestrogen- and/or progesterone-receptor status of tumour, histological diagnosis (WHO: ductal, lobular, tubular and medullar carcinoma or other malignant tumours), malignancy grading of ductal carcinomas (I–III according to mitosis activity, nuclear pleomorphy and degree of tubule formation; I being the most benign and III the most aggressive) and distant metastases at the time of surgery (evaluated by clinical examination in combination with chest X-ray and blood tests, including blood cell count and liver enzymes). The treatment-related variables were the intended adjuvant treatment according to trial protocols (radiotherapy, chemotherapy and endocrine treatment).

The medical records for all the augmented breast cancer cases, with the exception of one, which could not be found, and one randomly selected control per case were retrieved to obtain information on how the cancer was diagnosed, and details on the surgical procedure including implant status. We also compared the data reported to the DBCG register with the medical records. Generally, there was a high level of concordance in the reported data, but with regard to the variable ‘*tumour cells in the surgical margins*’ four augmented breast cancer women were recategorised based on the medical record information; in two cases, the biopsy specimen had been reported instead of the final specimen, in one case, the parameter was missing in the DBCG, but was found in the medical record, and in the last case, there were free margins, although narrow, and the patient was recategorised as having free margins. None of the 23 control subjects was recategorised regarding this variable, since there was complete agreement between the medical records and the DBCG-data.

### Statistical analyses

Frequencies were calculated for all variables. The differences between the women with breast implants (cases) and women from the background population (controls) in binary variables were evaluated with *χ*^2^ tests, and crude odds ratios (OR) and corresponding 95% confidence intervals (CI) were calculated. The difference in tumour size between the two groups was evaluated by analysis of variance. In order to preserve power in the tests, the matching variables were not included as covariates, because of the small number of breast cancer cases with cosmetic breast implants and the large range of values of these matching variables. All tests were performed with a 5% level of significance. Overall survival rates, including death from all causes, were estimated by the Kaplan–Meier method and a log-rank test was used for comparisons between the groups. Disease-free survival was not evaluated, as information on first recurrence was unavailable for the seven cases and 52 controls who did not participate in a postoperative treatment protocol.

## RESULTS

In total, 23 of 2955 women (0.8%) with cosmetic breast implants developed breast cancer after implantation. The mean age at the time of breast cancer diagnosis among these women as well as controls was 47.2 years, range 35–75. The mean year of cancer diagnosis was 1993, range 1986–2000. The women had undergone breast implantation at an average age of 38 years (range 22–61 years), yielding an average interval from implantation to breast cancer diagnosis of 9.3 years (range 0.3–17.9 years). A total of 21 women had silicone breast implants, while implant type was unknown for the remaining two, but they most likely also had silicone implants, since this was the preferred implant in Denmark during the relevant period. The majority of women in the study were premenopausal at the time of breast cancer diagnosis, which was to be expected in this relatively young study population. Fewer of the cases were premenopausal compared to the controls, although the difference was not significant (61 and 74%, respectively, OR=0.5; 95% CI 0.2–1.3).

As presented in Table 1

**Table 1:** Breast cancer tumour characteristics of women with cosmetic silicone breast implants prior to cancer diagnosis (cases) and breast cancer cases from the background population (controls)

	**Cases (*n*=23)**		**Controls (*n*=253)**		
**Characteristic of woman or tumour**	***n***	**%**	***n***	**%**	**OR (95% CI)^a^**
*Tumour histology (WHO)*					
Ductal	16	70	212	84	0.5 (0.2–1.6)^b^
Lobular	5	22	18	7	*P*=0.26
Other	0	0	18	7	
Unknown	2	9	5	2	
					
*Tumour hormone-receptor status*					
Negative	6	26	52	21	1.7 (0.6–4.9)
Positive	10	43	148	59	*P*=0.32
Unknown	7	30	53	21	
					
*Tumour diameter (mm) (mean, range)*	20.5	Range 9–50	25.2	Range 3–110	*P*=0.48
Unknown	3	13	10	4	
					
*Ductal carcinomas, malignancy grade (dedifferentiation)*					
Grade I	6	26	52	21	1.7 (0.6–5.0)^c^
Grade II	9	39	110	43	*P*=0.31
Grade III	1	4	40	16	
Nonductal or unknown	7	30	51	20	
					
*Breast cancer operation*					
Mastectomy	15	65	184	73	0.6 (0.3–1.6)^d^
Lumpectomy	6	26	60	24	*P*=0.34
Lumpectomy followed by mastectomy	0	0	4	2	
Biopsy only	2	9	5	2	
					
*Tumour cells in surgical margins (DBCG-data)*					
Yes	7	30	24	9	4.2 (1.5–11.4)
No	14	61	201	79	*P*=0.003
Unknown	2	9	28	11	
					
*Tumour cells in surgical margins (medical record information)*^e^					
Yes	5	22	24	9	2.5 (0.8–7.3)
No	17	74	201	79	*P*=0.09
Unknown	1	4	28	11	
					
*Axillary surgery*					
Five or less lymph nodes removed	4	17	40	16	1.2 (0.4–3.6)
More than five lymph nodes removed	18	78	210	83	*P*=0.79
Unknown	1	4	3	1	
					
Mean number of lymph nodes removed	11.4	Range 0–32	11.3	Range 0–31	*P*=0.16
					
*Axillary lymph node metastases*					
Node-negative	11	48	130	51	0.9 (0.4–2.2)
Node-positive	11	48	120	47	*P*=0.86
Unknown	1	4	3	1	
					
Mean number of lymph nodes affected	2.7	Range 0–20	2.2	Range 0–24	*P*=0.10
					
Women with distant metastases at time of diagnosis	1	4	4	2	*P*=0.36

a Women with implants (cases) compared with women from the background population (controls). Women with missing information with regard to the variable under study were excluded from the analysis.

b Ductal carcinomas *vs* all other cancers.

c Malignancy grade I *vs* malignancy grade II and III grouped together.

d Mastectomy *vs* lumpectomy. In case of conversion from lumpectomy to mastectomy, the category was mastectomy. In cases of biopsy only, the category was lumpectomy.

e Based on medical record information for 22 cases and 23 controls. For the remainder, the DBCG-data were used.

, most of the tumours were ductal carcinomas, and either receptor positive or with unknown receptor status, with no significant differences between the two groups. Tumour diameter tended to be larger among controls than among cases, but the difference was not significant. Tumour dedifferentiation, evaluated by grade of malignancy, did not differ between the two groups. The majority of women among both cases and controls were treated with mastectomy, which reflected the general treatment approach in Denmark during the period. Based on the DBCG-data, the women with breast implants were four times more likely than women in the control group to have tumour cells in the surgical margins (OR=4.2; 95% CI 1.5–11.4). Using the medical record data on surgical margins rather than the DBCG-data altered the statistical inference; the difference between cases and controls diminished and became nonsignificant (OR=2.5; 95% CI 0.8–7.3). Lymph node dissection was performed equally between implant women and controls. In total, 48% of the women in both groups had regional lymph node involvement and also to a similar extent. One patient in the implant group had distant metastases at the time of diagnosis compared with four in the control group (*P*=0.36).

As shown in Table 2

**Table 2:** Adjuvant treatment according to DBCG-protocols of women with cosmetic silicone breast implants prior to cancer diagnosis (cases) and women from the background population (controls)

	**Cases (*n*=23)**	**Controls (*n*=253)**			
**Adjuvant treatment according to protocol^a^**	***n***	**%**	***n***	**%**	**OR (95% CI)^b^**
*Subsequent adjuvant treatment (any)*					
No	6	26	63	24	1.2 (0.4–3.2)
Yes	14	61	175	69	*P*=0.73
Unknown	3	13	15	6	
					
*Subsequent chemotherapy*					
No	11	48	98	39	2.3 (0.8–6.9)
Yes	5	22	103	41	*P*=0.12
Unknown	7	30	52	21	
					
*Subsequent endocrine therapy*					
No	11	48	157	62	0.6 (0.2–1.9)
Yes	5	22	44	17	*P*=0.39
Unknown	7	30	52	21	
					
*Subsequent radiotherapy*					
No	10	43	129	51	0.8 (0.3–1.8)
Yes	12	52	119	47	*P*=0.56
Unknown	1	4	5	2	

a See The DBCG Secretariat (1998).

b Women with implants (cases) compared with women without implants (controls). Women with missing information with regard to the variable under study were excluded from the analysis.

, there was no difference in intended adjuvant treatment between the two groups. According to standard adjuvant treatment protocols, 61% of the women with breast implants and 69% of the controls were allocated to adjuvant treatment (Table 2). Approximately 50% of all patients among both cases and controls were scheduled for radiotherapy, including those with nonradical surgical margins. There were differences, although not significant, in systemic adjuvant treatment, with more implant women receiving endocrine therapy and more controls receiving chemotherapy. This pattern most likely reflects the differences in menopausal status, which influence the choice of treatment, since premenopausal women are more likely to receive chemotherapy.

**Figure 1:**
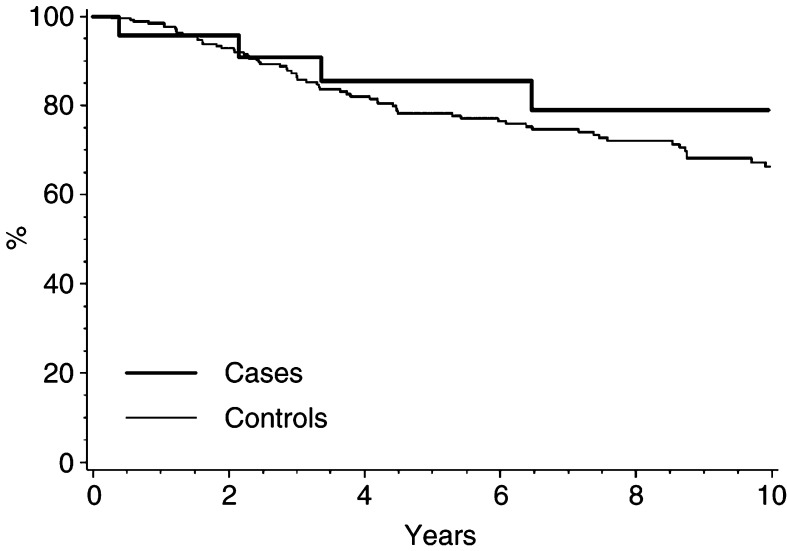
Overall survival after breast cancer in 23 women with cosmetic breast implants prior to cancer diagnosis (cases) and 253 breast cancer cases from the background population (controls).

In the survival analyses, the follow-up time was on average 6.4 years (range 0.3–15.7 years). Four women with breast implants died (17%), while 66 (26%) of the control subjects died. As illustrated in a Kaplan–Meier plot (Figure 1

), the 5-year survival estimate for implant women is 86% compared with 78% among the controls (*P*=0.36). The medical records stated that in 13 (57%) of the 23 breast cancer cases with implants, the implant was removed during the cancer surgery and in eight women (35%) it was left *in situ*. In one case, the implant had been removed prior to the cancer diagnosis, and in another case the medical records could not be traced. All five of the implant women who, according to the medical records, had tumour cells in the surgical margins were treated with mastectomy, and four of them also had explantation; for the last case this information was not available. The nonradical margins were found in the final mastectomy-specimen, and the women accordingly received postoperative adjuvant radiotherapy. Two of the implant women were treated with skin-sparing mastectomy, one kept her implant and the other was reconstructed with an immediate TRAM flap, but neither of these patients was among those with nonradical margins.

From the medical records we ascertained that 16 (70%) of the implant women discovered the tumour themselves. The remaining were diagnosed as follows: two were diagnosed clinically by physicians during diagnostic work-up because of other diseases, one was found by a plastic surgeon in a consultation related to capsular contracture, two were diagnosed after a biopsy performed during an operation for a presumed breast infection, and two were unknown. An equal proportion of the control subjects whose medical records were retrieved had found the tumour themselves. None of the augmented women with breast cancer and a potential maximum of five controls participated in mammography screening for breast cancer, evaluated by knowledge of the mammography screening centres active in the relevant areas and calendar period.

## DISCUSSION

The results of our study of breast cancer among women with cosmetic breast implants indicate that the cancer was not diagnosed later or at a more advanced stage than in the normal population. The women with augmented breasts in our study did not differ from controls with regard to relevant tumour characteristics at diagnosis. More of the women with implants had tumour cells in the surgical margins, but we observed no evidence of an unfavourable survival within our rather limited follow-up time.

Clinical case series have reported a tendency towards a delayed breast cancer diagnosis among women with breast implants (Gottlieb *et al*, 1984; Leibman and Kruse, 1990; Silverstein *et al*, 1990a; Carlson *et al*, 1993; Fajardo *et al*, 1995). Overall, epidemiological studies have not been able to confirm such reports. Two large cohort studies (Birdsell *et al*, 1993; Deapen *et al*, 2000), which compared breast implant women with breast cancer to breast cancer cases in the background population, reported findings similar to ours. There were no significant differences with regard to stage of disease at diagnosis or survival. Information on the cases and controls was obtained from medical records and cancer registers, respectively, so information bias cannot be ruled out. Also in concordance with our findings, a clinical study (Cahan *et al*, 1995) comparing 22 augmented breast cancer patients with all other breast cancer patients in the same surgical clinic found no differences in stage of disease at diagnosis. It was concluded that nonpalpable and preinvasive breast cancer can be detected in the augmented patient. A similar study (Clark *et al*, 1993) of all breast cancer cases in augmented women diagnosed at the study clinic found that augmented women tended to have smaller tumours and more often their tumours were diagnosed at physical examination (by themselves or their physician) than other breast cancer patients diagnosed at the clinic. Significantly fewer of the augmented women had nodal involvement (19 *vs* 41% in the comparison group). However, in both the above studies, it was not stated how the patients were referred to the clinic, and selection bias may have influenced the results. In another cohort study (Brinton *et al*, 2000b), 78 cases of breast cancer in women with breast implants were compared with breast cancer cases in a control cohort of women who had undergone other kinds of cosmetic surgery. A tendency towards more advanced stages in cases compared with controls was reported. However, the differences were not statistically significant. As in our study, no differences in overall survival were noted.

The presence of breast implants, silicone or saline, makes mammography more difficult (Gumucio *et al*, 1989). The implant obscures some of the parenchyma, and breasts with subglandular positioned implants with capsular contracture are the most difficult to examine (Carlson *et al*, 1993). Specific techniques have been developed to increase the efficacy of mammography among augmented women (Eklund *et al*, 1988). A methodological study (Silverstein *et al*, 1990b) compared preoperative mammograms with postaugmentation mammograms in 62 healthy women, and found that the implants obscured from 9 to 44% of the glandular tissue, depending on implant location, mammography view and technique. Obtaining a baseline mammography prior to and perhaps even after implantation has been recommended (Leibman and Kruse, 1990; Silverstein *et al*, 1992).

Overall, the results of the above mammographic and epidemiological studies point in different directions regarding delayed breast cancer diagnosis among implanted women. Since most of the women in our study found the tumour themselves, a potential impairment of mammography could not be evaluated. It is possible that in a population of women undergoing regular breast cancer screening with mammography, women with breast implants could be diagnosed at a later state than women without implants. None of the studies to date has sufficient data to answer this question. However, the presence of breast implants, especially submuscularly positioned implants, often makes both clinical examination and self-examination of the breast tissue easier, since the glandular tissue can be palpated against a firm background, and this may account for the lack of delay in diagnosis (Clark *et al*, 1993). Women who receive breast implants tend to focus on health issues and be self-attentive (Anderson, 1998), and to have less breast tissue, thus probably making them more likely to find a breast tumour even at an early stage.

Both cases and controls in our study received on average the same surgical and adjuvant treatment. However, more implant women had residual tumour cells in the surgical margins, even though this excess was not statistically significant when based on information from the medical records. Medical record information is likely to be more complete than the DBCG register data, but was only obtained for a small subset of the controls, and this could introduce selection bias. If we had reviewed the medical records from all the background controls, which was not feasible because of problems with the availability of the records, we may have encountered controls who would qualify for reclassification, and this could have affected our calculations in an unknown direction.

We had an *a priori* expectation that women with implants would be more likely to request breast preservation than other women and speculated that this could account for the excess of women with nonradical margins. However, similar proportions of women in the two groups were treated with mastectomy, including all the women with nonradical margins. Free surgical margins are a strong predictor of both local control of the disease and survival (Clemons *et al*, 2001; Medina-Franco *et al*, 2002); however, cases did not have a poorer survival than controls. The average follow-up time of 6.4 years is limited with respect to evaluating survival, since death from breast cancer can occur several years after the cancer diagnosis, and the study population is small, so the results should be interpreted with caution. To our knowledge, no other study has reported on the risk of nonradical tumour resection among women with breast implants, and this issue needs further attention.

We found few cases of breast cancer in the cohort of women with cosmetic breast implants. Previous findings based on the same cohort (Mellemkjaer *et al*, 2000) identified 16 cases of breast cancer among 2740 women with cosmetic breast implants under 55 years of age. Compared to the 17.3 expected cases, this yielded a standardised incidence ratio (SIR) of 0.9 (95% CI 0.5–1.5). Others have reported similar findings and concluded that women with cosmetic breast implants are not at increased risk of subsequent breast cancer (Berkel *et al*, 1992; Deapen *et al*, 1997; McLaughlin *et al*, 1998; Brinton *et al*, 2000b); on the contrary, a recent meta-analysis reported a 30% reduction of breast cancer risk among women with breast implants (RR=0.72; 95% CI 0.61–0.85) and it was concluded that breast implants may confer a protective effect against breast cancer (Hoshaw *et al*, 2001). Several explanations for this protective effect have been put forward: activation of the immune response as result of foreign body reaction may enhance detection and degradation of precancerous lesions; compression of the glandular tissue from the implant may diminish blood perfusion, which may alter cellular metabolism, and a local decrease in body temperature caused by the implant could diminish cellular metabolism (Deapen *et al*, 1997; Brinton *et al*, 2000b; Hoshaw *et al*, 2001). However, different preoperative characteristics among women seeking breast implantation compared with other women could perhaps more likely account for the decreased breast cancer risk among implant women. This includes less use of oral contraception (Kjøller *et al*, 2003), less alchohol use (Fryzek *et al*, 2000; Kjøller *et al*, 2003), lower age at first birth, higher parity, and lower BMI (Cook *et al*, 1997; Brinton *et al*, 2000a; Fryzek *et al*, 2000; Kjøller *et al*, 2003). Amount of glandular tissue may also play a role although this has been highly debated, mostly because of the problems of evaluating the true glandular mass of the breast (Hsieh and Trichopoulos, 1991; Tavani *et al*, 1996; Thurfjell *et al*, 1996; Egan *et al*, 1999) However, in both Swedish and Danish investigations of women who underwent breast reduction surgery, large statistically significant reduction in breast cancer incidence of 30–50% were observed, respectively (Boice *et al*, 1997,^2000^). In a recent study based on the same Swedish cohort of women with breast hypertrophy, a reduction in breast cancer risk proportionate to the amount of resected breast tissue was reported (Brinton *et al*, 2001). These studies support the concept of the amount of breast tissue being a risk factor in breast cancer.

The primary limitations of our study are the small number of breast cancer cases among augmented women and the relatively short follow-up time, which suggest that survival estimates be interpreted with caution. Potential bias exists, since more augmented breast cancer cases were treated outside adjuvant protocols, and there may be differences in the quality of information between the two groups, which thus may not be as comparable as intended. However, since information was sampled from the same registries, this potential bias is less of a concern than in other studies in which different data sources were used. The strengths of this study include the ability to have virtually complete follow-up of a well-defined cohort of women with cosmetic breast implants and to obtain highly detailed clinical and staging information for both cases and controls from the DBCG-register, which permitted evaluation of numerous important tumour- and treatment-related parameters. In addition, linkage with the Danish Cancer Register allowed complete ascertainment of all breast cancer cases.

In conclusion, the present study suggests, consistent with several other epidemiological studies, that the stage of breast cancer at diagnosis in breast-implanted woman does not appear to be more advanced compared with age- and calendar-matched controls, indicating that the diagnosis is not meaningfully delayed because of the implants, and that overall survival appears not to be influenced by the presence of implants. We suggest that the issue of nonradical surgical margins be investigated in future studies.
